# Feeling Unsafe at School Among Adolescents in 13 Asian and European Countries: Occurrence and Associated Factors

**DOI:** 10.3389/fpsyt.2022.823609

**Published:** 2022-04-25

**Authors:** Yuko Mori, Elina Tiiri, Lotta Lempinen, Anat Brunstein Klomek, Gerasimos Kolaitis, Helena R. Slobodskaya, Hitoshi Kaneko, Jorge C. Srabstein, Liping Li, Mai Nguyen Huong, Samir Kumar Praharaj, Say How Ong, Sigita Lesinskiene, Henriette Kyrrestad, Tjhin Wiguna, Zahra Zamani, Lauri Sillanmäki, Andre Sourander, the EACMHS Study Group, Shahin Akhondzadeh

**Affiliations:** ^1^Department of Child Psychiatry, University of Turku, Turku, Finland; ^2^INVEST Research Flagship Center, University of Turku, Turku, Finland; ^3^Department of Child Psychiatry, Turku University Hospital, University of Turku, Turku, Finland; ^4^Baruch Ivcher School of Psychology, Reichman University, Herzliya, Israel; ^5^Department of Child Psychiatry, School of Medicine, Aghia Sophia Children's Hospital, National and Kapodistrian University of Athens, Athens, Greece; ^6^Department of Medicine, Scientific Research Institute of Neuroscience and Medicine, Novosibirsk, Russia; ^7^Department of Medicine, Novosibirsk State University, Novosibirsk, Russia; ^8^Psychological Support and Research Center for Human Development, Nagoya University, Nagoya, Japan; ^9^Division of Psychiatry and Behavioral Sciences, Children's National Hospital, Washington, DC, United States; ^10^Department of Psychiatry and Behavioral Sciences, School of Medicine, George Washington University, Washington, DC, United States; ^11^Shantou University Medical College, Shantou, China; ^12^Department of Psychiatry, Vietnam National Children's Hospital, Hanoi, Vietnam; ^13^Department of Psychiatry, Kasturba Medical College, Manipal, India; ^14^Manipal Academy of Higher Education, Manipal, India; ^15^Department of Developmental Psychiatry, Institute of Mental Health, Singapore, Singapore; ^16^Vilnius University, Faculty of Medicine, Institute of Clinical Medicine, Clinic of Psychiatry, Vilnius, Lithuania; ^17^Faculty of Health Sciences, Regional Centre for Child and Youth Mental Health and Child Welfare, UiT the Arctic University of Norway, Tromsø, Norway; ^18^Department of Psychiatry, Faculty of Medicine, Dr. Cipto Mangunkusumo General Hospital, Universitas Indonesia, Jakarta, Indonesia; ^19^Department of Community Medicine, Tehran University of Medical Sciences, Tehran, Iran

**Keywords:** school safety, school climate, mental health, adolescent, associated factors, feeling unsafe, cross-national comparisons, occurrence

## Abstract

**Background:**

Research on perceived school safety has been largely limited to studies conducted in Western countries and there has been a lack of large-scale cross-national studies on the topic.

**Methods:**

The present study examined the occurrence of adolescents who felt unsafe at school and the associated factors of perceived school safety in 13 Asian and European countries. The data were based on 21,688 adolescents aged 13-15 (11,028 girls, 10,660 boys) who completed self-administered surveys between 2011 and 2017. Logistic regression analyses were used to estimate odds ratios and 95% confidence intervals.

**Findings:**

The number of adolescents who felt unsafe at school varied widely across countries, with a mean occurrence of 31.4% for the total sample: 31.3% for girls, and 31.1% for boys. The findings revealed strong independent associations between feeling unsafe and individual and school-related factors, such as being bullied, emotional and behavioral problems and feeling that teachers did not care. The study also found large variations in perceived school safety between schools in many countries.

**Conclusion:**

The findings emphasize the need to create safe educational environments for all students, based on positive relationships with teachers and peers. School-based interventions to prevent bullying and promote mental health should be a natural part of school safety promotion.

## Introduction

A safe school environment is essential for the educational success and development of children and young people. The United Nations' Educational, Scientific and Cultural Organization defines school safety as the process of establishing and maintaining a school that provides a physically, cognitively, and emotionally safe space where students and staff can carry out learning activities (1).

Perceived school safety has mainly been studied in western countries. A recent systematic review of 43 studies showed the mean occurrence of unsafe school environments was ~19% and ranged from 6.1 to 69.1% (2). Feeling safe at school has been reported to be associated with various predictors, including bullying and youth violence (3, 4), lower academic achievements (5) and better relationships with teachers (6). Feeling unsafe may have a negative impact on mental health, which may persist throughout life. It has been associated with mental health issues among adolescents, such as depressive symptoms, suicidal behavior and self-harm (7, 8).

Although school safety is often included in studies as one of the aspects of a school environment, a recent systematic review reported a lack of consensus on what constituted a school climate (9). School climate has been reported to be associated with socioemotional and behavioral outcomes (10). However, it is not clear whether perceived school safety in itself accounts for these associations or if they can be explained by other aspects of a school climate, such as the institutional environment.

According to the systematic review, most of the studies on perceived school safety were published between 2016 and 2020, indicating an increasing research interest in this topic (2). However, very few cross-national studies have been conducted and all of these only compared perceived school safety between two countries (11–13). In addition, there is no well-established definition of school safety (14). The impact of feeling unsafe at school on mental health outcomes, and the association with surrounding environments, such as school characteristics, has been understudied (15, 16). This study contributes to this growing area of research by exploring perceived school safety in 13 countries using the same study method in all of the countries.

This study explored perceived school safety in 13 Asian and European countries. The first aim was to report the cross-national comparisons of the occurrence of adolescents feeling unsafe at school in 13 Asian and European countries. The second aim was to assess the associations between perceived school safety and individual factors (e.g., sex and emotional and behavioral difficulties) and school-related factors (e.g., school type and location). We also wanted to see whether there were any variations in the probability of feeling unsafe in different schools in each country. This was the first large-scale cross-national study to examine the occurrence of school safety and its associations with individual and school characteristics.

## Methods

The Eurasian Child Mental Health Study group is a large, international study body that conducts cross-national research on the wellbeing and mental health of children and adolescents (17). This study was part of the Eurasian Child Mental Health Study and comprised 13 countries: China, Finland, Greece, India, Indonesia, Iran, Israel, Japan, Lithuania, Norway, Russia, Singapore, and Vietnam.

### Sample

We surveyed 28,427 adolescents in the 13 countries between 2011 and 2017. The median response rate was 88.9% and varied from 51.7% in Indonesia to 97.1% in Iran. Because there were variations in the age ranges in the total samples across countries, we focused on adolescents aged 13-15 years to make the data more comparable. After the age restriction, a total of 21,688 adolescents (50.8% girls) from 200 schools were included. Their mean age was 13.9 years and the mean number of schools in the 13 countries was 16. The mean number of participants was 1,679 and ranged from 946 in Vietnam to 2,988 in Finland. The survey year and the characteristics of each country's study sample are presented in Table 1.

**Table 1:** Demographic characteristics of the 13 countries.

**Country**	**Survey year**	**Total** ***N***	**Sex (girl)** ***N*** **(%)**	**Age** **Mean (SD) [range]**	**School location (urban)** ***N*** **(%)**	**School type (public)** ***N*** **(%)**	**Number of schools**
China	2016	2,119	1040 (49.1)	13.8 (0.8) [13-15]	819 (36.8)	1,779 (79.9)	10
Finland	2014	2,982	1493 (50.1)	14.1 (0.8) [13-15]	2,686 (89.9)	2,988 (100)	13
Greece	2016	1,040	556 (53.5)^*^	13.6 (0.6) [13-15]	750 (72.1)	1,040 (100)	14
India	2016	1,672	864 (51.7)	13.6 (0.7) [13-15]	1,420 (84.9)	209 (12.5)	11
Indonesia	2016	1,023	542 (53.0)	13.5 (0.6) [13-15]	1,024 (100)	656 (64.1)	5
Iran	2016	1,178	557 (47.3)	14.3 (0.8) [13-15]	1,178 (100)	1,036 (87.9)	16
Israel	2014	1,277	698 (54.7)^*^	14.0 (0.8) [13-15]	1,101 (100)	1,246 (97.4)	10
Japan	2011	1,828	943 (51.6)	13.9 (0.3) [13-14]	833 (45.5)	1,831 (100)	17
Lithuania	2016	2,507	1256 (50.1)	14.1 (0.8) [13-15]	1,353 (53.8)	2,515 (100)	17
Norway	2017	1,900	946 (49.8)	13.9 (0.8) [13-15]	1,611 (84.8)	1,742 (99.4)	45
Russia	2015	1,051	546 (52.0)	14.1 (0.8) [13-15]	1,051 (100)	1,051 (100)	20
Singapore	2014	2,165	1103 (50.9)	14.0 (0.8) [13-15]	2,165 (100)	2,165 (100)	24
Vietnam	2016	946	484 (51.2)	13.9 (0.8) [13-15]	946 (100)	946 (100)	3

*The chi-square test for equal proportions was used to analyze sex distribution*.

Asterisk (*) indicates statistical significance, p < 0.05.

### Questionnaire and Procedure

The current study was conducted using a self-administered survey, which was based on a questionnaire previously used among adolescents in Finland (18, 19). The questionnaires were translated into the local language and back-translated for accuracy (20). All students at school at the time of the survey were invited to participate and completed the questionnaires during school hours. The questionnaires were collected by the teachers in 11 countries and returned to the researchers and were completed electronically in Norway and Singapore.

### Ethics

The researchers obtained ethical approval from the Institutional Review Boards in their countries and obtained permission from the schools. Participation was voluntary and anonymity was guaranteed. Consent was obtained from the parents or school authorities, according to each country's policies. The study was performed in accordance with the ethical standards laid down in the 1964 Declaration of Helsinki and its later amendments.

### Measures

School safety was assessed by a single item: “I feel safe at school.” The possible answers were never, sometimes, often and always. Because the never category was very small in some countries, such as 2.2% in Finland, we combined the responses into binary outcomes. We did this by pooling never and sometimes and then often and always. This enabled us to compare the different countries. The a priori reference category for the 13 countries was Finland, as it had the lowest occurrence of adolescents who felt unsafe at school. The adolescents' ages were subdivided into 13, 14, and 15 years of age and the reference category was 13 years old. Gender was dichotomized into girls and boys.

To assess bullying victimization, a definition of bullying was provided: “A student is getting bullied, if another student or a group of students repeatedly treats him/her negatively or in an insulting manner. It is difficult for the bullied student to defend himself/herself. Bullying can be intermittent or continuous. Bullying can be verbal (e.g., calling names, threatening), physical (e.g., hitting, pushing) or psychological (e.g., spreading rumors, avoiding, excluding). Continuous nasty or insulting teasing is also bullying.” Cyberbullying was defined as: “Repeated mocking on the Internet, bullying via emails or text messages or spreading insulting material about another person on the Internet.” The students were asked, “How often have you been bullied in school in the past 6 months?,” “How often have you been bullied away from school in the past 6 months?” for traditional bullying and the options were never, less than once a week, more than once a week, and most days. Cyberbullying was measured by asking “During the past 6 months, how often have you been cyberbullied?” and the options were never, less than once a week, more than once a week, and almost every day. For all bullying questions, we combined the responses into binary outcomes: no for never and yes for the other responses. After that, we combined these variables and compared adolescents who were not bullied, who were only traditionally bullied, who were only cyberbullied, and who were both traditionally and cyberbullied.

Emotional and behavioral difficulties were assessed with a self-report version of the Strengths and Difficulties Questionnaire (SDQ). Five subscales measured emotional symptoms, conduct problems, hyperactivity, peer problems and prosocial behavior and each had five items (21). Because our study sample represented the general population, we followed Goodman's advice and used the broader internalizing subscales, covering emotional symptoms and peer problems, and the externalizing subscales, covering conduct problems and hyperactivity symptoms. The adolescents were asked if they had overall difficulties with their emotions, concentration, behavior or getting along with other people. This question is part of the SDQ impact supplement and was thought to provide useful further insight into psychiatric cases and the need for health services. The possible answers were no, yes minor difficulties, yes definite difficulties or yes severe difficulties. These were coded as no, mild, moderate and severe difficulties and combined into three categories: no versus mild vs. moderate and severe.

Their views on teachers were measured by a single item: “Teachers care about me.” The possible answers were never, sometimes, often and always. We combined never and sometime and often and always.

School characteristics were assessed by the type and location of the school. Researchers from each country selected a mixture of rural and urban and public and privately funded study schools. The reference categories were public schools for type and urban schools for location.

### Statistical Analysis

The responses from all countries were pooled to create various descriptive statistics. First, an unadjusted univariate logistic regression was conducted to identify any association between school safety and explanatory variables. Significant interactions (p < 0.1) were found between sex and other explanatory variables: country, bullying victimization, SDQ externalizing symptoms, perceived difficulties and whether teachers cared. Sex-specific analysis and reporting are encouraged in health research to understand the implications of the differences for preventive intervention and treatment following the raising awareness of sex difference in health and women's underrepresentation or exclusion in research data (22, 23). Therefore, further analyses were conducted separately for sex. Second, the regression analyses were adjusted for age and country. Third, the regression analyses were adjusted for all the other explanatory variables. The multivariate analyses were performed using two models, because information about perceived difficulties were missing from Japan and Israel. We used multivariable regression analysis to adjust for all the explanatory variables, except perceived difficulties (model one). Then we included perceived difficulties, but excluded Israel and Japan (model two). Finally, a logistic regression model was used to estimate the probabilities of feeling unsafe in different schools in each country. This was done separately for girls and boys including school as an explanatory variable and adjustment was made for age. We included 200 schools and the mean number of schools in the 13 countries was 16. If a school had <10 girls or boys who felt unsafe at school, it was excluded from the analyses for this sex. Two-sided p-values of <0.05 were considered statistically significant, with the exception of the interaction analysis (p < 0.1). The statistical analyses used SAS 9.4 for Windows (SAS Institute Inc. Cary, NC, USA).

## Results

Table 2 summarizes the responses and explanatory variables from the 13 countries. The occurrence of adolescents who felt unsafe at school, based on the different categories of explanatory variables and the results of the regression analysis, are shown separately for girls (Table 3) and boys (Table 4). This shows that 31.4% of the total sample felt unsafe and the occurrence was 31.3% for the girls and 31.1% for the boys. The interaction between safety and sex was significant for several explanatory variables, namely country, bullying victimization, SDQ externalizing subscale, whether teachers cared and perceived difficulties.

**Table 2:** Summary of responses and explanatory variables in the 13 participating countries.

**Variables**	**N**	**Mean**	**%**	**SD**
**School safety**				
Always/often	14,749	..	68.6	..
Never/sometimes	6,739	..	31.4	..
**Sex**				
Girls	11,028	..	50.8	..
Boys	10,660	..	49.2	..
**Age**				
13	6,953	..	31.9	..
14	9,147	..	41.9	..
15	5,722	..	26.2	..
**Bullying victimization**				
Not victimized	15,256	..	71.9	..
Traditional only	3,722	..	17.5	..
Cyber only	1,005	..	4.7	..
Combined	1,238	..	5.8	..
**Emotional and behavioral difficulties**				
Externalizing	..	6.22	..	3.09
Internalizing	..	5.51	..	3.40
**Perceived difficulties**				
No	8825	..	44.7	..
Mild	8140	..	41.2	..
Moderate/severe	2787	..	14.1	..
**Teacher care**				
Always/often	11950	..	55.9	..
Never/sometimes	9442	..	44.1	..
**School type**				
Public	19204	..	88.6	..
Private	2464	..	11.4	..
**School location**				
Urban	16937	..	78.3	..
Rural	4700	..	21.7	..

*SD, standard deviation*.

**Table 3:** Univariate and multivariate analyses of the explanatory variables associated with feeling unsafe at school among girls.

**Variables**	**Category**	**Never/sometimes safe**		**Univariate analysis**	**Multivariate analysis**	
					**Model 1^a^**	**Model 2^b^**
		***N*** **(%)**	**Mean (SD)**		**OR (95%CI)**			
Sex	Girl	3,415 (31.3)	..	..	..	..
Country	Finland	171 (11.5)	..	Reference	Reference	Reference
	Norway	131 (13.9)	..	1.24 (0.97-1.58)	1.96 (1.48-2.61)^*^	1.88 (1.41–2.51)^*^
	Israel	101 (14.6)	..	1.31 (1.00-1.70)^*^	1.31 (0.94-1.82)	..
	Greece	101 (18.2)	..	1.70 (1.30-2.23)^*^	3.02 (2.22–4.11)^*^	3.01 (2.21–4.12)^*^
	India	153 (17.8)	..	1.66 (1.31–2.10)^*^	3.33 (2.32–4.80)^*^	3.67 (2.53–5.31)^*^
	Iran	139 (25.6)	..	2.64 (2.06–3.39)^*^	2.35 (1.76–3.13)^*^	2.46 (1.84–3.29)^*^
	Indonesia	164 (30.3)	..	3.33 (2.61–4.24)^*^	3.74 (2.79–5.00)^*^	3.74 (2.79–5.02)^*^
	Lithuania	387 (31.4)	..	3.50 (2.87–4.28)^*^	3.45 (2.73–4.37)^*^	3.65 (2.87–4.65)^*^
	Singapore	390 (35.4)	..	4.21 (3.43–5.15)^*^	5.50 (4.36–6.94)^*^	5.65 (4.46–7.15)^*^
	China	499 (48.8)	..	7.30 (5.97–8.93)^*^	11.03 (8.58–14.17)^*^	11.59 (8.95–15.01)^*^
	Russia	294 (54.7)	..	9.24 (7.32–11.66)^*^	9.05 (6.93–11.82)^*^	9.09 (6.94–11.92)^*^
	Vietnam	239 (49.6)	..	7.54 (5.94–9.58)^*^	7.91 (6.03–10.39)^*^	8.28 (6.27–10.94)^*^
	Japan	646 (69.8)	..	17.76 (14.36–21.96)^*^	20.15 (15.58–26.07)^*^	..
Age	13	1,024 (28.5)	..	Reference	Reference	Reference
	14	1,647 (35.7)	..	1.39 (1.27–1.53)^*^	0.92 (0.81–1.04)	0.94 (0.83–1.07)
	15	744 (27.3)	..	0.94 (0.84–1.05)	0.88 (0.76–1.01)	0.91 (0.79–1.05)
Bullying victimization	Not victimized	2,085 (26.5)	..	Reference	Reference	Reference
	Traditional only	725 (43.3)	..	2.11 (1.90–2.36)^*^	1.83 (1.59–2.10)^*^	1.83 (1.58–2.12)^*^
	Cyber only	191 (35.7)	..	1.54 (1.28–1.85)^*^	1.53 (1.23–1.91)^*^	1.50 (1.19–1.89)^*^
	Combined	340 (52.3)	..	3.04 (2.58–3.57)^*^	2.70 (2.20–3.31)^*^	2.55 (2.06–3.15)^*^
Emotional and behavioral difficulties	Externalizing	..	7.07 (3.03)	1.60 (1.54–1.67)^*^	1.06 (1.04–1.08)^*^	1.06 (1.03–1.08)^*^
	Internalizing	..	7.35 (3.61)	1.72 (1.65–1.80)^*^	1.10 (1.08–1.12)^*^	1.09 (1.07–1.11)^*^
Perceived difficulties	No	703 (18.1)	..	Reference	Reference	Reference
	Mild	1,378 (32.0)	..	2.31 (2.08–2.57)^*^	..	1.18 (1.04–1.34)^*^
	Moderate/Severe	665 (38.9)	..	3.56 (3.11–4.08)^*^	..	1.55 (1.29–1.86)^*^
Teacher care	Always/Often	893 (14.4)	..	Reference	Reference	Reference
	Never/Sometimes	2,499 (53.5)	..	6.82 (6.22–7.47)^*^	5.35 (4.81–5.95)^*^	5.14 (4.58–5.77)^*^
School type	Private	302 (26.1)	..	Reference	Reference	Reference
	Public	3,103 (32.1)	..	1.33 (1.16–1.53)^*^	0.88 (0.68–1.13)	0.90 (0.70–1.16)
School location	Urban	2,430 (28.5)	..	Reference	Reference	Reference
	Rural	959 (42.2)	..	1.84 (1.67–2.02)^*^	1.07 (0.92–1.23)	1.05 (0.89–1.23)

*Differences in the numbers of participants between tables are due to missing information*.

Asterisk (*) indicates statistical significance (p < 0.05).

*SD, standard deviation; OR, odds ratio; CI, confidence interval*.

a *Adjusted for all explanatory variables, except perceived difficulties*.

b *Adjusted for all explanatory variables, excluding Japan and Israel*.

**Table 4:** Univariate and multivariate analyses of the explanatory variables associated with feeling unsafe at school among boys.

**Variables**	**Category**	**Never/sometimes safe**		**Univariate analysis**	**Multivariate analysis**	
					**Model 1^a^**	**Model 2^b^**
		**N (%)**	**Mean (SD)**		**OR (95%CI)**			
Sex	Boy	3,252 (31.1)	..	..	..	..
Country	Finland	131 (8.9)	..	Reference	Reference	Reference
	Norway	73 (7.7)	..	0.85 (0.63–1.15)	1.42 (1.02–1.99)^*^	1.41 (1.01–1.98)^*^
	Israel	81 (14.2)	..	1.68 (1.25–2.26)^*^	1.52 (1.06–2.17)^*^	..
	Greece	116 (24.2)	..	3.25 (2.47–4.28)^*^	4.59 (3.36–6.28)^*^	4.75 (3.46–6.51)^*^
	India	209 (26.3)	..	3.63 (2.86–4.62)^*^	6.95 (4.98–9.70)^*^	7.24 (5.17–10.14)^*^
	Iran	211 (34.3)	..	5.33 (4.17–6.81)^*^	4.22 (3.21–5.56)^*^	4.19 (3.18–5.53)^*^
	Indonesia	145 (30.2)	..	4.40 (3.38–5.74)^*^	5.78 (4.23–7.90)^*^	5.56 (4.05–7.64)^*^
	Lithuania	425 (35.4)	..	5.58 (4.50–6.92)^*^	4.35 (3.40–5.57)^*^	4.58 (3.56–5.87)^*^
	Singapore	363 (34.3)	..	5.32 (4.27–6.63)^*^	6.78 (5.28–8.69)^*^	6.73 (5.23–8.65)^*^
	China	457 (44.1)	..	8.04 (6.47–9.99)^*^	10.43 (8.01–13.57)^*^	11.36 (8.67–14.89)^*^
	Russia	220 (45.9)	..	8.67 (6.73–11.18)^*^	7.43 (5.57–9.91)^*^	7.43 (5.55–9.94)^*^
	Vietnam	243 (52.6)	..	11.33 (8.77–14.63)^*^	9.98 (7.46–13.35)^*^	9.92 (7.38–13.33)^*^
	Japan	578 (68.2)	..	21.93 (17.42–27.61)^*^	21.86 (16.60–28.77)^*^	..
Age	13	890 (27.7)	..	Reference	Reference	Reference
	14	1,561 (36.1)	..	1.47 (1.33–1.62)^*^	1.02 (0.90–1.16)	1.02 (0.89–1.16)
	15	801 (27.6)	..	0.99 (0.89–1.11)	0.97 (0.84–1.11)	0.99 (0.85–1.14)
Bullying victimization	Not victimized	1,939 (27.1)	..	Reference	Reference	Reference
	Traditional only	805 (40.3)	..	1.82 (1.64–2.02)^*^	1.67 (1.47–1.90)^*^	1.65 (1.44–1.89)^*^
	Cyber only	152 (33.2)	..	1.34 (1.10–1.64)^*^	1.08 (0.86–1.37)	1.03 (0.81–1.31)
	Combined	265 (46.7)	..	2.37 (1.99–2.81)^*^	2.04 (1.66–2.52)^*^	1.98 (1.59–2.48)^*^
Emotional and behavioral difficulties	Externalizing	..	7.19 (3.09)	1.49 (1.43–1.56)^*^	1.03 (1.02–1.05)^*^	1.03 (1.01–1.05)^*^
	Internalizing	..	6.05 (3.50)	1.67 (1.60–1.74)^*^	1.10 (1.08–1.12)^*^	1.11 (1.09–1.13)^*^
Perceived difficulties	No	1,086 (22.8)	..	Reference	Reference	Reference
	Mild	1,175 (31.9)	..	1.73 (1.57–1.91)^*^	..	1.00 (0.88–1.13)
	Moderate/Severe	388 (37.4)	..	2.46 (2.11–2.87)^*^	..	1.25 (1.03–1.52)^*^
Teacher care	Always/Often	853 (15.0)	..	Reference	Reference	Reference
	Never/Sometimes	2,377 (50.7)	..	5.83 (5.32–6.40)^*^	5.42 (4.86–6.04)^*^	5.53 (4.91–6.21)^*^
School type	Private	368 (28.5)	..	Reference	Reference	Reference
	Public	2,878 (31.7)	..	1.16 (1.02–1.32)^*^	1.28 (1.03–1.58)^*^	1.30 (1.05–1.61)^*^
School location	Urban	2,336 (28.6)	..	Reference	Reference	Reference
	Rural	896 (41.0)	..	1.74 (1.58–1.92)^*^	1.06 (0.92–1.22)	0.94 (0.80–1.10)

*Differences in the numbers of participants between tables are due to missing information*.

Asterisk (*) indicates statistical significance (p < 0.05).

*SD, standard deviation; OR, odds ratio; CI, confidence interval*.

a *Adjusted for all explanatory variables, except perceived difficulties*.

a *Adjusted for all explanatory variables, excluding Japan and Israel*.

Tables 3, 4 provide separate odds ratios (ORs) by sex for the explanatory variables. When we adjusted the data for age and country, the same variables from the unadjusted univariate logistic regression remained significant, except school location for boys. In multivariate model one, a significant association was noted between feeling unsafe and just being traditionally bullied (girls OR 1.83, 95% CI 1.59-2.10 and boys OR 1.67, 95% CI 1.47-1.90) and combined traditional and cyberbullying (girls OR 2.70, 95% CI 2.20-3.31 and boys OR 2.04, 95% CI 1.66-2.52). Cyberbullying was significantly associated with feeling unsafe for girls but not for boys (OR 1.53, 95% CI 1.23-1.9). When it came to emotional and behavioral difficulties, there were significant associations between feeling unsafe and both internalizing behavior (girls OR 1.10, 95% CI 1.08-1.12 and boys 1.10, 95% CI 1.08-1.12) and externalizing behavior (girls OR 1.06, 95% CI 1.04-1.08 and boys OR 1.03, 95% CI 1.02-1.05). The same was true if they felt their teachers were less caring (girls OR 5.35, 95% CI 4.81-5.95 and boys OR 5.42, 95% CI 4.86-6.04). Most countries reported higher odds for feeling unsafe than the reference country, Finland, except Israeli girls. The greatest odds were in Japan (girls OR 20.15, 95% CI 15.58-26.07 and boys OR 21.86, 95% CI 16.60-28.77).

The same variables from model one remained significant in model two. A significant association was also found between feeling unsafe and moderate and severe perceived difficulties (girls OR 1.18, 95% CI 1.04-1.34 and boys OR 1.00, 95% CI 0.88-1.13). However, only girls had mild perceived difficulties (OR 1.55, 95% 1.29-1.86). There were no significant associations between age and feeling unsafe in both multivariate models.

School type and location were not significantly associated with girls feeling unsafe after adjustment. Public schools remained associated with boys feeling unsafe throughout the analyses (OR 1.30, 95% 1.05-1.61), but no significant association was found with school location after the data were adjusted.

Because a strong association was found between feeling unsafe and combined traditional and cyberbullying, a *post-hoc* analysis examined whether this combined victimization more strongly related to feeling unsafe than traditional bullying only and cyberbullying only. This revealed that combined victimization was more strongly associated with feeling unsafe than traditional bullying only (girls OR 0.59, 95% CI 0.49-0.72 and boys OR 0.74, 95% CI 0.60-0.90) or cyberbullying only (girls OR 0.46, 95% CI 0.36-0.60 and boys OR 0.53, 95% CI 0.40-0.69). The same additional analysis was conducted on perceived difficulties and this revealed that moderate or severe perceived difficulties were more strongly associated with feeling unsafe than mild perceived difficulties (girls OR 1.54 95% CI 1.36-1.75 and boys OR 1.42, 95% CI 1.22-1.66).

**Figure 1:**
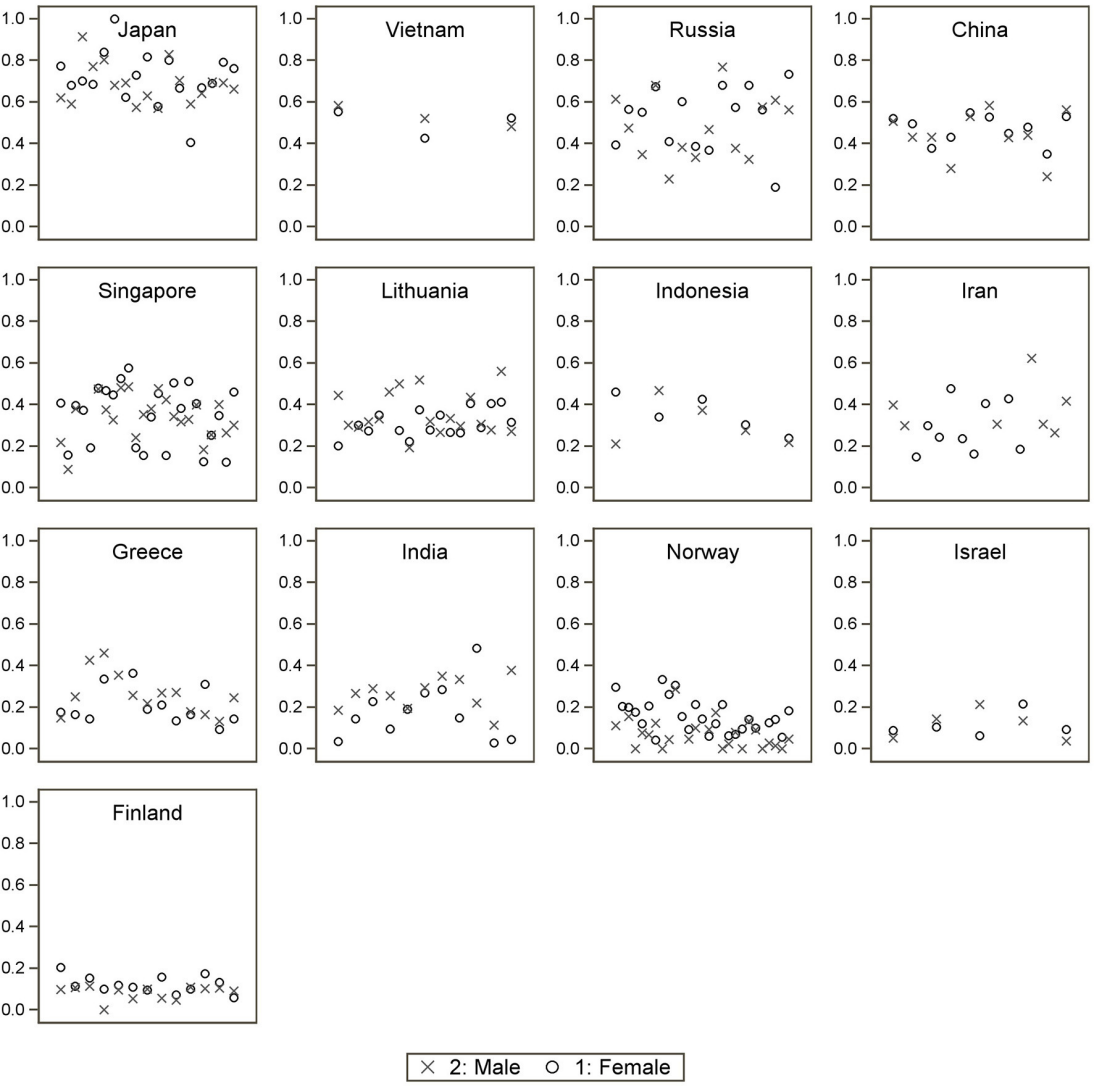
Predicted probabilities of feeling unsafe at school by school and country, adjusted for age. The X axis shows schools in random order.

Figure 1 shows the predicted probabilities of feeling unsafe at different schools, by country and sex. For girls, the range in predicted probabilities of feeling unsafe between schools was smallest in Vietnam (0.43-0.55, mean 0.5, range 0.13) and largest in Japan (0.41-1.00, mean 0.72, range 0.59). For boys, it was smallest in Vietnam (0.48-0.58, mean 0.53, range 0.10) and largest in Russia (0.23-0.77, mean 0.48, range 0.54).

## Discussion

This study had three key findings. First, a striking proportion of approximately 30% of the adolescents did not feel safe at school and there were large variations across the 13 Asian and European countries, from 11.5% in Finland to 69.8% in Japan for girls and from 7.7% in Norway to 68.2% in Japan for boys. Second, a strong independent association was found between feeling unsafe and individual and school-related factors, such as being bullied and emotional and behavioral difficulties. Third, there were large variations in the probability of perceived school safety between schools in many countries.

The fact that nearly one-third of adolescents did not feel safe at school suggests that violence and insecurity in society could be highly associated with these fears. Finland and Norway are Nordic welfare states and the occurrence of adolescents who felt unsafe was very low. In contrast, Japanese adolescents were most likely to feel unsafe, despite living in a highly developed country with significantly low crime rates and a comparatively modest social safety net (24, 25). The reasons for these variations were unclear, but they may reflect cultural and social differences.

Adolescents were more likely to feel unsafe at school if they lived in countries with greater power distance and collectivistic cultures. Power distance is the degree to which countries accept the hierarchy of power and collectivism refers to a value that emphasize on conformity to group harmony rather than individual interests (26). A previous study of 31 countries found that adolescents who lived in such countries felt less connected with school communities than students who lived in countries that focused on individuals and had more equal power (27). Teacher-centered and strictly disciplined education within cultures high in power distance may inhibit positive teacher-student relationships, in contrast to cultures where teachers and students are perceived as more equal (28). Moreover, the highly competitive and stressful environment in Asian schools may reduce feelings of safety (29), as excessive academic distress has been linked with emotional and behavioral difficulties (30). In addition, corporal punishment is still legal in one-third of the countries in the world and students could feel less safe if they received or witnessed physical punishment at school (31).

### Individual Factors Associated With Feeling Unsafe

This was the first large scale cross-national study to assess whether being victimized by traditional bullying, cyberbullying or the combination of these was associated with feeling unsafe at school. We found that both traditional victimization and combined victimization were strongly associated with feeling unsafe for both sexes. However, cyberbullying victimization was only associated with feeling unsafe in girls, who are generally more likely to be cyberbullied than boys (32).

We found that both internalizing and externalizing symptoms were significantly associated with feeling unsafe. In addition, moderate and severe perceived difficulties were independently associated with feeling unsafe in both sexes. Longitudinal studies have shown that both witnessing violence and victimization at school predicted later internalizing and externalizing problems (33, 34). Individuals with mental health difficulties may start feeling unsafe because they perceive a lack of social support or stigma (35). However, the mechanisms between mental health symptoms and feeling unsafe need clarification. For example, bullying victimization has been associated with both emotional and behavioral difficulties and feeling unsafe at school but the causes and directions of these are unknown (4, 36).

We did not find any significant differences between the age groups, in contrast to previous studies that found differences in perceived school safety between younger and older students (11, 37). This may be explained by the limited age range of 13-15 years in our study, compared to wider age ranges in other studies.

### School Factors Associated With Feeling Unsafe

The fact that both sexes felt unsafe if they perceived that teachers did not care was a striking finding. In contrast, good teacher-student relationships, with secure attachments, have been associated with increased psychosocial wellbeing and reduced mental health issues (38). In our study, boys were more likely to feel unsafe at public, than private schools, but no difference was found in girls. Physical conflicts and gang-related activity tend to be lower in private schools (39). Furthermore, bullying and physical aggression are more common among boys (40), which may explain why girls were not affected.

### Variations in the Probability of Feeling Unsafe in Different Schools by Country

We found variations in the probability of feeling unsafe in different schools in the 13 countries. In some countries, like Finland, the variations were quite small and this could reflect the homogenous quality of education across the country (41). Private schools often require high tuition fees, compared to Government-funded schools, which may contribute to the variation between schools, especially in countries that included private schools. Our findings emphasize the importance of providing safe educational environments for all students, regardless of their background or the schools they attend.

## Strengths and Limitations

The strength of our study included the use of same measures from large number of countries. The same definition of bullying and cyberbullying were provided in all countries. However, there were some limitations. First, the surveys were conducted in certain regions of those countries. Therefore, the generalisability of the results is subject to certain limitations. For instance, the occurrence rate refers to occurrence in that geographical area which participated in the study and may not represent the whole country. This lack of sample representativeness is a common methodological issue in cross-national research (42). However, we aimed to include all the schools in specific geographical area in the countries to increase the representativeness. Second, we tried to select public and private schools in both urban and rural locations. However, the sample largely consisted of private schools in countries like India and Indonesia and some countries, like Finland, did not include any private schools. This discrepancy was partially due to the different educational systems. For example, in Finland, there are only few private schools whereas, in some countries, these are the mainstream. Additionally, the number of schools we included on the studies varies across countries. This may have affected the representativeness of the study. Third, we did not have data on classroom teachers such as their qualification and length of experience in the education which are also known to be associated with school climate and bullying (43, 44). Fourth, it is possible that some meanings were slightly changed when the questionnaires were translated, such as the definitions of safety. Cultural factors may also have influenced some of the variation in the occurrencebecause the interpretation of concepts such as feeling of safety or bullying can differ across cultures and languages. In addition, the restriction of the survey to adolescents currently attending school and present on the day of the survey may have also led to some underreporting of occurrence of students who feel unsafe at school as bullying and perceived lack of safety at school are associated with higher risk of absenteeism (45). Lastly, the cross-sectional study design was purely observational, and no causal inference can be drawn from the findings.

## Conclusion

Nearly one-third of the adolescents we studied felt unsafe at school, which was really striking and creates a challenge for societies. Safe educational environments are based on building care and trust with teachers and promoting positive interactions with others, rather than being socially isolated. They can also create a backdrop for positive developments in adolescence and prevent bullying. This makes school safety a critical issue for both educational systems and public health. Adolescents who experienced mental health difficulties or were bullied were more likely to feel unsafe at school. This emphasizes the need for school-based, anti-bullying interventions and mental health promotion. These should include initiatives such as psychoeducation, and social–emotional learning programs to prevent behavioral problems and enhance the prosocial competence of all children. Our findings highlight inequality in securing a safe educational environment for students within, and among, countries. There is clearly a need for strategies to promote educational environments where all students can feel safe and be protected.

## Data Availability Statement

The raw data supporting the conclusions of this article will be made available by the authors, without undue reservation.

## Ethics Statement

The studies involving human participants were reviewed and approved by the Institutional Review Boards in each countries by the researchers and obtained permission from the schools (e.g., The Ethics Committee at the University of Turku in Finland). Written informed consent to participate in this study was provided by the participants' legal guardian/next of kin.

## The Eacmhs Study Group

Shahin Akhondzadeh, Psychiatric Research Center, Roozbeh Hospital, Tehran University of Medical Sciences, Iran; Daniel S. S. Fung, Department of Developmental Psychiatry, Institute of Mental Health, Singapore; George Giannakopoulos, Department of Child Psychiatry, School of Medicine, National and Kapodistrian University of Athens, Aghia Sophia Children's Hospital, Athens, Greece; Meytal Grimland, Shalvata Mental Health Center, Hod Hasharon, Israel; the Department of Epidemiology and Preventive Medicine, Sackler Faculty of Medicine, Tel Aviv University, Tel Aviv, Israel; Shoko Hamada, Department of Psycho-Social Studies, School of Arts and Letters, Meiji University, Tokyo, Japan; Roshan Chudal, Department of Child Psychiatry, University of Turku, Turku, Finland; Emmi Heinonen, Department of Child Psychiatry, University of Turku, Turku, Finland; Raden Irawati Ismail, Department of Psychiatry, Faculty of Medicine Universitas Indonesia-dr. Cipto Mangunkusumo General Hospital, Jakarta-Indonesia; Praveen A. Jain, Department of Psychiatry, Kasturba Medical College, Manipal, Manipal Academy of Higher Education, Manipal, Karnataka, India; Avinash G. Kamath, Department of Psychiatry, Kasturba Medical College, Manipal, Manipal Academy of Higher Education, Manipal, Karnataka, India; Jerrine Z. N. Khong, Advocacy and Research Department, Singapore Children's Society, Singapore; Sturla Fossum, Regional Centre for Child and Youth Mental Health and Child Welfare, Faculty of Health Sciences, UiT The Arctic University of Norway, Tromsø, Norway; Albert Prabowo Limawan, Faculty of Medicine Universitas Indonesia; Maryam Mohseny, Department of Community Medicine, Shahid Beheshti University of Medical Sciences, Tehran, Iran; Ali Najafi, Tehran University of Medical Sciences, Tehran, Iran; Ngoc Minh Thanh, Department of Psychiatry, Vietnam National Children's Hospital, Hanoi, Vietnam; Masayoshi Ogura, Graduate School of Education, Naruto University of Education, Tokushima, Japan; Zhekuan Peng, Shantou University Medical College, Shantou, China; Tatiana O. Rippinen, Research Institute of Neuroscience and Medicine, Novosibirsk, Russia; Rini Sekartini, Department of Child Health, Faculty of Medicine Universitas Indonesia-dr. Cipto Mangunkusumo General Hospital, Jakarta-Indonesia; Nadezhda B. Semenova, Scientific Research Institute of Medical Problems of the North, Krasnoyarsk, Russia; Norbert Skokauskas, the Regional Centre for Child and Youth Mental Health and Child Welfare, Faculty of Medicine and Health Sciences, Norwegian University of Science and Technology, Trondheim, Norway; Yi Ren Tan, Department of Developmental Psychiatry, Institute of Mental Health, Singapore; Kalliopi Triantafyllou, Department of Child Psychiatry, School of Medicine, National and Kapodistrian University of Athens, Aghia Sophia Children's Hospital, Athens, Greece; Foivos Zaravinos-Tsakos, Department of Child Psychiatry, School of Medicine, National and Kapodistrian University of Athens, Aghia Sophia Children's Hospital, Athens, Greece.

## Author Contributions

AS, AK, SO, HKY, HKA, GK, SL, LLI, MH, SP, LS, JS, HS, TW, and ZZ contributed to the general study conception and design. Study design and data collection in each country was performed by the authors in the participating countries. Data management and harmonization was performed by LS. Data analysis was planned by AS, YM, ET, LLE, and performed by LS. The first draft of the manuscript was written by YM, ET, LLE, LS, and AS. All authors including those in the EACMHS Study Group commented on previous versions of the manuscript. All authors read and approved the final manuscript.

## Funding

This study was funded by the Academy of Finland (decision numbers 320162 and 308552). The Russian arm of the study was supported by the Russian Science Foundation (grant number 21-15-00033).

## Conflict of Interest

The authors declare that the research was conducted in the absence of any commercial or financial relationships that could be construed as a potential conflict of interest.

## Publisher's Note

All claims expressed in this article are solely those of the authors and do not necessarily represent those of their affiliated organizations, or those of the publisher, the editors and the reviewers. Any product that may be evaluated in this article, or claim that may be made by its manufacturer, is not guaranteed or endorsed by the publisher.
