# Supplementary material for: Feeling Unsafe at School Among Adolescents in 13 Asian and European Countries: Occurrence and Associated Factors
Source: Front Psychiatry. 2022 Apr 25;13:823609. doi: 10.3389/fpsyt.2022.823609 (PMC9082541; doi:10.3389/fpsyt.2022.823609)
Supplement: Supplementary file 1 [file Table_1.DOCX]

Supplementary Material

# Supplementary Table 1 | Distribution of feeling safe at school among girls and boys by country.

| **Country** | **Sex** | **Never *N* (%)** | **Sometimes *N* (%)** | **Often *N* (%)** | **Always *N* (%)** |
| --- | --- | --- | --- | --- | --- |
| China | Girls | 100 (9·8) | 399 (39·0) | 242 (23·7) | 282 (27·6) |
|  | Boys | 137 (13·2) | 320 (30·9) | 278 (26·8) | 302 (29·1) |
| Finland | Girls | 30 (2·0) | 141 (9·5) | 431 (29·1) | 881 (59·4) |
|  | Boys | 35 (2·4) | 96 (6·5) | 428 (29·2) | 909 (61·9) |
| Greece | Girls | 20 (3·6) | 81 (14·6) | 150 (27·0) | 305 (54·9) |
|  | Boys | 36 (7·5) | 80 (16·7) | 121 (25·2) | 243 (50·6) |
| India | Girls | 27 (3·1) | 126 (14·7) | 68 (7·9) | 639 (74·3) |
|  | Boys | 63 (7·9) | 146 (18·3) | 132 (16·6) | 455 (57·2) |
| Indonesia | Girls | 24 (4·4) | 140 (25·8) | 170 (31·4) | 208 (38·4) |
|  | Boys | 37 (7·7) | 108 (22·5) | 143 (29·7) | 193 (40·1) |
| Iran | Girls | 50 (9·2) | 89 (16·4) | 124 (22·8) | 280 (51·6) |
|  | Boys | 96 (15·6) | 115 (18·7) | 137 (22·3) | 267 (43·4) |
| Israel | Girls | 20 (2·9) | 81 (11·7) | 88 (12·7) | 504 (72·7) |
|  | Boys | 27 (4·7) | 54 (9·4) | 94 (16·4) | 397 (69·4) |
| Japan | Girls | 320 (34·6) | 326 (35·2) | 202 (21·8) | 77 (8·3) |
|  | Boys | 310 (36·6) | 268 (31·6) | 181 (21·4) | 88 (10·4) |
| Lithuania | Girls | 111 (9·0) | 276 (22·4) | 307 (24·9) | 540 (43·8) |
|  | Boys | 219 (18·2) | 206 (17·1) | 240 (20·0) | 537 (44·7) |
| Norway | Girls | 41 (4·4) | 90 (9·6) | 147 (15·6) | 664 (70·5) |
|  | Boys | 34 (3·6) | 39 (4·1) | 118 (12·5) | 754 (79·8) |
| Russia | Girls | 102 (19·0) | 192 (35·7) | 111 (20·6) | 133 (24·7) |
|  | Boys | 81 (16·9) | 139 (29·0) | 110 (23·0) | 149 (31·1) |
| Singapore | Girls | 54 (4·9) | 336 (30·5) | 337 (30·6) | 374 (34·0) |
|  | Boys | 82 (7·7) | 281 (26·5) | 357 (33·7) | 339 (32·0) |
| Vietnam | Girls | 50 (10·4) | 189 (39·2) | 106 (22·0) | 137 (28·4) |
|  | Boys | 78 (16·9) | 165 (35·7) | 83 (18·0) | 136 (29·4) |
| Total | Girls | 949 (8·7) | 2466 (22·6) | 2483 (22·7) | 5024 (46·0) |
|  | Boys | 1235 (11·8) | 2017 (19·3) | 2422 (23·2) | 4769 (45·7) |
